# Endothelial cells from pulmonary endarterectomy specimens possess a high angiogenic potential and express high levels of hepatocyte growth factor

**DOI:** 10.1186/s12890-018-0769-3

**Published:** 2018-12-29

**Authors:** Akira Naito, Seiichiro Sakao, Irene M. Lang, Norbert F. Voelkel, Takayuki Jujo, Keiichi Ishida, Toshihiko Sugiura, Goro Matsumiya, Ichiro Yoshino, Nobuhiro Tanabe, Koichiro Tatsumi

**Affiliations:** 1 Department of Respirology, Graduate School of Medicine, Chiba University, 1-8-1 Inohana, Chuo-Ku, Chiba City, 260-8670 Japan; 2 Department of Advancing Research on Treatment Strategies for respiratory disease, Graduate School of Medicine, Chiba University, 1-8-1, Inohana, Chuo-Ku, Chiba City, 260-8670 Japan; 3 Department of Internal Medicine II, Cardiology, Medical University of Vienna, Spitalgasse 23, 1090 Vienna, Austria; 4 Victoria Johnson Center for Obstructive Lung Disease, Virginia Commonwealth University, 1101 East Marshall Street, Sanger Hall, Richmond, VA 23298-0565 USA; 5 Department of Advanced Medicine in Pulmonary Hypertension, Graduate School of Medicine, Chiba University, 1-8-1 Inohana, Chuo-Ku, Chiba City, 260-8670 Japan; 6 Department of Cardiovascular Surgery, Graduate School of Medicine, Chiba University, 1-8-1 Inohana, Chuo-Ku, Chiba City, 260-8670 Japan; 7 Department of Thoracic Surgery, Graduate School of Medicine, Chiba University, 1-8-1 Inohana, Chuo-Ku, Chiba City, 260-8670 Japan

**Keywords:** Chronic thromboembolic pulmonary hypertension, Pulmonary endarterectomy, Endothelial cell, Hepatocyte growth factor, Angiogenesis

## Abstract

**Background:**

Impaired angiogenesis is assumed to be an important factor in the development of chronic thromboembolic pulmonary hypertension (CTEPH). However, the role of endothelial cells (ECs) in CTEPH remains unclear. The aim of this study was to investigate the angiogenic potential of ECs from pulmonary endarterectomy (PEA) specimens.

**Methods:**

We isolated ECs from PEA specimens (CTEPH-ECs) and control EC lines from the intact pulmonary arteries of patients with peripheral lung cancers, using a MACS system*.* These cells were analyzed in vitro including PCR-array analysis, and the PEA specimens were analyzed with immunohistochemistry. Additionally, the serum HGF levels were determined in CTEPH patients.

**Results:**

A three-dimensional culture assay revealed that CTEPH-ECs were highly angiogenic. An angiogenesis-focused gene PCR array revealed a high expression of hepatocyte growth factor (HGF) in CTEPH-ECs. The high expression of HGF was also confirmed in the supernatant extracted from PEA specimens. The immunohistochemical analysis showed expression of HGF on the surface of the thrombus vessels. The serum HGF levels in CTEPH patients were higher than those in pulmonary thromboembolism survivors.

**Conclusion:**

Our study suggests that there are ECs with pro-angiogenetic character and high expression of HGF in PEA specimens. It remains unknown how these results are attributable to the etiology. However, further investigation focused on the HGF pathway may provide novel diagnostic and therapeutic tools for patients with CTEPH.

**Electronic supplementary material:**

The online version of this article (10.1186/s12890-018-0769-3) contains supplementary material, which is available to authorized users.

## Background

Chronic thromboembolic pulmonary hypertension (CTEPH) is a form of pulmonary hypertension that is related to unresolved organizing clots and it is generally acknowledged that pulmonary endarterectomy (PEA) is the gold standard for CTEPH treatment. Various etiological factors, including infection, inflammation, genetic susceptibilities, and insufficient angiogenesis [1], have been discussed as important pathogenetic factors [2]. Poor angiogenesis detected in PEA tissue specimens also implies a poor prognosis in CTEPH patients [3]. In addition, disordered angiogenesis which leads to the delayed resolution of thrombus may be a potential etiological factor underlying the development of CTEPH [4]. However, it remains to be elucidated whether endothelial cells (ECs) are involved in the pathogenesis of this disease because of the limited number of investigations that have been reported.

Hepatocyte growth factor (HGF) and its receptor Mesenchymal-epithelial transition factor (Met), which was originally found to be a potent mitogen that promotes hepatocyte growth and liver regeneration [5, 6], are now proposed to have prominent roles in the cardiovascular diseases. It is reported that HGF has pleiotropic functions, including proliferation, angiogenesis, anti-apoptosis, anti-autophagy, anti-inflammation and anti-fibrosis in the vascular ECs, myocardial cells, and other types of cells [7], however, the precise role of HGF- Met signaling in the etiology of CTEPH remains unknown.

Here we wish to elucidate the angiogenesis-related characteristics of ECs harvested from PEA specimens from a viewpoint of HGF.

## Methods

### Study population

CTEPH patients who underwent PEA from June 2014 to October 2015 were enrolled. PEA specimens were obtained from CTEPH patients during PEA performed by Dr. Ishida at Chiba University Hospital. The process by which CTEPH was diagnosed has been described previously [8]. During the same period, pulmonary arteries were obtained at Chiba University Hospital from lung lobectomy specimens of patients with stage I peripheral lung cancer. The diagnosis of stage I lung cancer was confirmed by chest enhanced computed tomography, brain magnetic resonance imaging and histological examination of the lung tissue. No patients received chemotherapy or radiotherapy prior to surgery, and patients with evident chronic obstructive pulmonary disease or pulmonary fibrosis were excluded from the study.

### Tissue collection

At the time of PEA, pieces of PEA specimens contained in the segmental pulmonary arteries were collected. Segmental pulmonary artery samples of approximately 1.5 cm in length were collected from lung lobectomy specimens; these arteries were located far from the primary tumor and did not show macroscopic or microscopic malignant cell invasion. These arteries were collected for primary cell culture and histopathological examination in the same manner as PEA specimens.

### Isolation of CTEPH-ECs and control-ECs

The PEA specimens and pulmonary arteries from lung lobectomy specimens were washed in sterile phosphate buffered saline (PBS), minced, and incubated in Dulbecco’s Modified Eagle Medium (DMEM) with 1% bovine serum albumin, 0.2% collagenase (Wako, Tokyo, Japan), 10 mg/ml DNaseI (Wako, Tokyo, Japan), 250 mg/ml dispase (Roche, Tokyo, Japan) for 1 h. The obtained cell suspensions were seeded into a 6-cm-petri dish coated with fibronectin (Corning, Corning, NY, USA) and incubated in an endothelial growth medium™-2 microvascular BulletKit™ (EGM; Lonza, Basel, Switzerland) until they reached approximately 80% confluence (7–10 days) at 37 °C in 5% CO_2_ in an humidified air incubator. At the first passage, CD 31-positive ECs were isolated using CD31 MicroBeads (Miltenyi Biotec, Tokyo, Japan), as described previously [9]. The cells were incubated in EGM, and all of the experiments were carried out using cells that had been passaged less than 6 times.

### Immunocytochemistry

The cells were fixed in 4% paraformaldehyde for 10 min followed by blocking with PBS-T (PBS with Tween-20, 0.1%) containing 2% normal goat serum for 30 min, and incubated with a primary antibody overnight at 4 °C and with a secondary antibody for 1 h at room temperature. The stained cells were washed with 4 μg/ml DAPI in PBS for 5 min, embedded in 50% glycerin and examined with a Fluoview FV10i-LIV (Olympus, Tokyo, Japan).

### The cell growth curve, permeability assay and three-dimensional culture

To assess the proliferative potential, 5 × 10^4^ ECs were seeded in a 6-cm Petri dish. At each indicated day, the cells were trypsinized and counted. We also evaluated the permeability of each EC cell line with CultreCoat® In Vitro Vascular Permeability Assay (Trevigen, Inc. MD, USA) according to the manufacturer’s instructions. Matrigel™ Basement Membrane Matrix (Corning, Corning, NY, USA) was used for the three-dimensional culture in accordance with the manufacturer’s instructions. Additional details have been described previously [10]. The total tube lengths in the microscopic fields (taken a Nikon ECLIPSE Ti-S, Tokyo, Japan; capture magnification, × 40) were calculated using the Image J software program (ver. 1.48, National Institutes of Health, Bethesda, MD; http://imagej.nih.gov/ij/).

### Total RNA isolation from ECs and the PCR-based angiogenesis-related gene analysis

Total RNA was extracted from CTEPH-ECs and Control-ECs with RNeasy Mini Kit (Qiagen, Tokyo, Japan) according to the manufacturer’s instruction. RT2-Profiler™ PCR Arrays (Qiagen, Tokyo, Japan) were used to analyze the expression of a focused panel of genes involved in various biological processes. The Human Angiogenesis (PAHS-024Z) 96-well plate, which profiles the expression of 84 key genes that are involved in angiogenesis, was selected to detect the differential expression of genes between CTEPH-ECs and Control-ECs. The detailed method has been described previously [9].

### HGF inhibition in proliferating CTEPH-ECs and three-dimensional culture

To investigate the effects of HGF inhibition in cultured CTEPH-ECs, we used tivantinib, a selective HGF/MET inhibitor (Selleck, Houston, TX, USA) [11]. Similarly to the studies that examine the cell growth curve and the three-dimensional culture experiments that are detailed above, 5 × 10^4^ CTEPH-ECs were seeded in a 6-cm Petri dish with EGM containing 1 μM tivantinib or 0.1% DMSO (vehicle). At each indicated day, the cells were trypsinized and counted. Furthermore, 5 × 10^4^ of CTEPH-ECs were seeded on Matrigel™ Basement Membrane Matrix with EGM containing 1 μM tivantinib or 0.1% DMSO (vehicle) and incubated at 37 °C for 24 h, as described above.

### Total RNA isolation from PEA specimens and control pulmonary arteries and the PCR-based angiogenesis-related gene analysis

Total RNA was extracted from PEA specimens and control pulmonary arteries with RNeasy Fibrous Tissue Mini Kit (Qiagen, Tokyo, Japan) according to the manufacturer’s instruction. RT^2^ qPCR Primer Assays (Qiagen, Tokyo, Japan) were used to analyze the expression of HGF and other inflammatory molecules.

### Protein extraction from PEA specimens and Western blotting

The extraction and quantification of the protein from tissues (PEA specimens and pulmonary arteries) [12] have been described previously. Protein samples (10 μg) were separated on NuPAGE® Novex 10% Bis-Tris Gel (Invitrogen, Tokyo, Japan) and transferred to nitrocellulose membranes (Invitrogen, Tokyo, Japan). Membranes were blocked with 5% non-fat dried milk in PBS containing 0.5% Tween20 for 1 h at room temperature, and were then incubated with primary antibodies overnight at 4 °C. The membranes were incubated with peroxidase-conjugated secondary antibodies for 1 h at room temperature. Chemiluminescence was detected using a LAS-4000 (Fuji Film, Tokyo, Japan). The blots were scanned and a densitometry analysis was conducted using the Image J software program.

### Immunohistochemistry

Samples were fixed in 10% buffered formalin, paraffinized and cut into 3 μm-thick slices. Deparaffinized sections were washed with PBS and blocked with PBS-T containing 2% normal goat serum for 30 min at room temperature. They were then incubated with the primary antibodies overnight at 4 °C and with secondary antibodies for 1 h at room temperature. Stained slices were washed with 4 μg/ml DAPI in PBS for 5 min, embedded in 50% glycerin and examined with a Fluoview FV10i-LIV.

### Reagents

The following antibodies (Abs) were used in immunocytometry and immunohistochemistry: rabbit anti-von Willebrand factor (Factor VIII) (1:200, DAKO, Carpinterria, CA, USA), mouse anti-CD31 Ab (1:200, Abcam, Tokyo, Japan), mouse anti-Vimentin (1:200, Dako, CA, USA), mouse anti-Desmin (1:100, DAKO, Carpinterria, CA, USA), rabbit anti-hepatocyte growth factor (HGF) Ab (1:200, Abcam, Tokyo, Japan), rabbit anti-Met Ab (1:200, Abcam, Tokyo, Japan), normal Rabbit IgG isotype control (1:200, R&D Systems, Minneapolis, MN, USA), goat anti-rabbit IgG conjugated with Alexa-488 fluorescent dye (1:200, Thermofisher Scientific, Tokyo, Japan) and goat anti-mouse IgG conjugated with Alexa-594 fluorescent dye (1:200, Thermofisher Scientific, Tokyo, Japan). These antibodies were dissolved in PBS-T.

The following Abs were used in Western blotting: rabbit anti-βactin (1:1000, Biolegend, Tokyo, Japan), rabbit anti-HGF (1:1000, Abcam, Tokyo, Japan) and Goat anti-Rabbit IgG HRP conjugate (1:1000, Thermoscientific, Tokyo, Japan). These antibodies were dissolved with blocking solution (5% non-fat dried milk in PBS containing 0.5% Tween20).

### Blood samples

Blood serum samples were collected from CTEPH patients at the time of their diagnostic right heart catheterization from August 2013 to June 2017. Control serum samples were obtained from pulmonary thromboembolism (PTE) survivors without evidence of pulmonary hypertension during a routine medical consultation more than 3 months after the acute PTE episode. The serum level of HGF was measured by enzyme immunoassay method (LSI Medience Inc.; Tokyo, Japan).

### Statistical analysis

The PCR-array data were analyzed by the RT^2^ profiler PCR array Data Analysis web-based software program (https://www.qiagen.com/jp/shop/genes-and-pathways/data-analysis-center-overview-page/), and the fold regulations are expressed as the mean and the 95% confidence interval. Other statistical analyses were performed with commercially available software (GraphPad Prism, version 6.0.2, San Diego, CA) and the results are expressed as mean ± standard deviation (SD) unless otherwise described. Serum HGF levels were analyzed using Welch’s t-test and paired t-test. Other statistical analyses were compared using Mann-Whitney test. *P* values of < 0.05 were considered to indicate statistically significant differences.

## Results

### Cell culture and characterization of EC

**Fig. 1:**
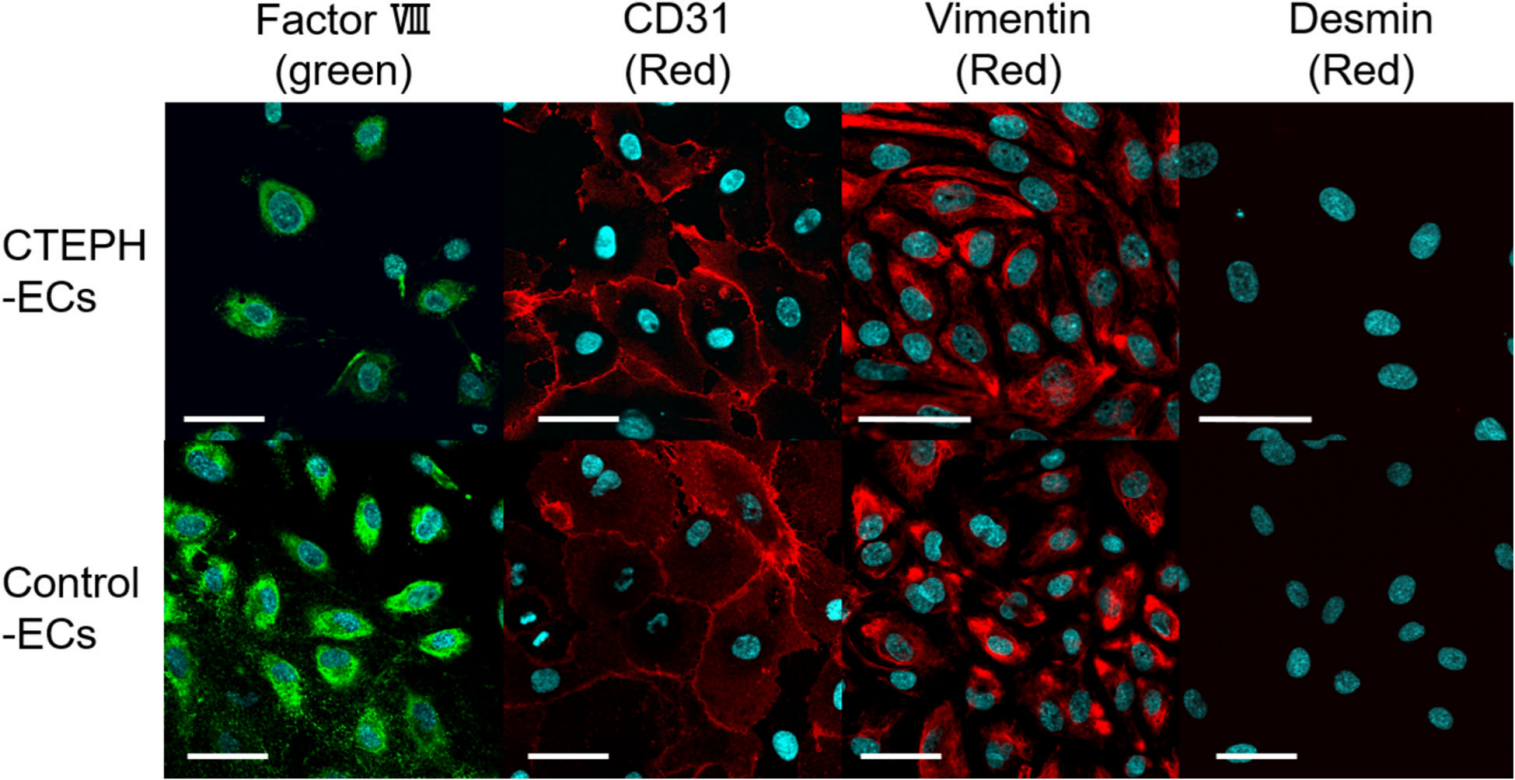
Immunocytochemistry of the cells from pulmonary endarterectomy specimens (CTEPH-ECs) and from the human pulmonary arteries (Control-ECs). Both CTEPH-ECs and Control-ECs were positive for Factor VIII, CD31, vimentin and negative for desmin (DAPI staining [blue]; Bar = 50 μm)

Endothelial cells from five CTEPH patients and three lung cancer patients were isolated (We were unable to purify ECs from the other specimens because of contamination by fibroblast-like cells). Immunocytochemistry revealed that the CTEPH-ECs and control-ECs were positive for CD31, Factor VIII, vimentin and negative for desmin (Fig. 1).

### The high proliferative and angiogenic potential of CTEPH-ECs

**Fig. 2:**
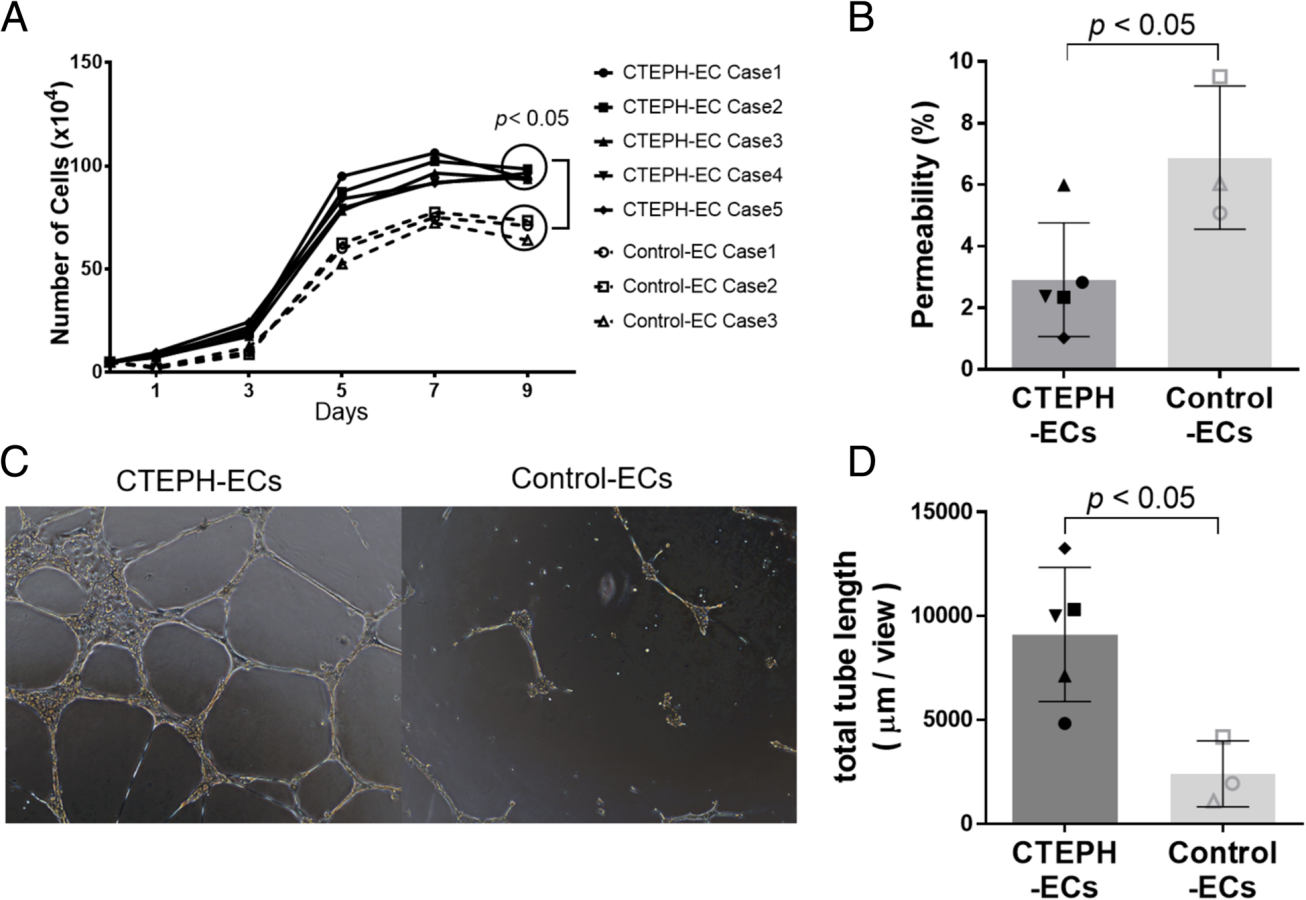
The proliferation and angiogenic potential of CTEPH-ECs. **a** The cell growth curve showed the greater proliferative potential of CTEPH-ECs. **b** Permeability assay of each cell line. The value is expressed as a percentage to the well without cells (100% permeability). **c**, **d** Three-dimensional culture showed that CTEPH-ECs made much more tubes than Control-ECs

Each CTEPH-EC line showed a greater degree of proliferation than the control-ECs in the cell growth curve analysis (day 9; *p* = 0.035) (Fig. 2a) and CTEPH-ECs had lower permeability compared to Control-ECs (*p* = 0.035) (Fig. 2b). In the three-dimensional culture experiments, the CTEPH-ECs showed a greater degree of tube formation (Fig. 2c), and the total tube lengths were statistically longer in comparison to the control-ECs (*p* = 0.035) (Fig. 2d).

### The high expression of HGF in CTEPH-ECs

**Fig. 3:**
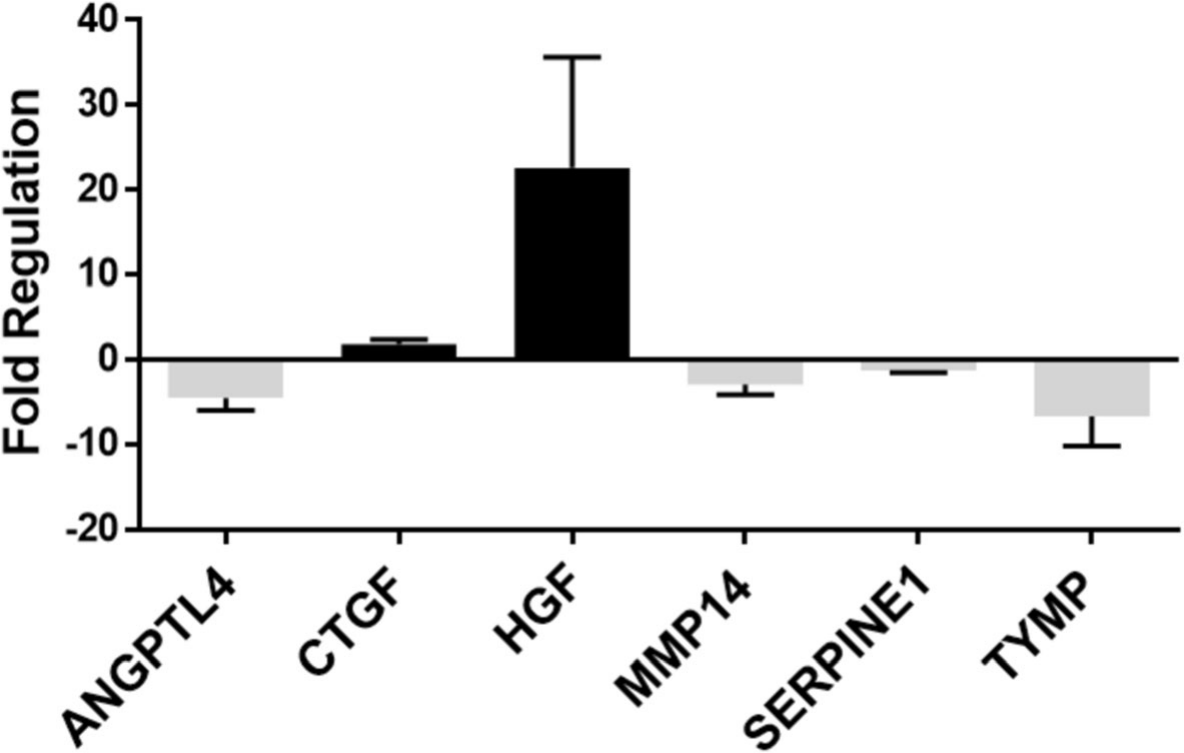
PCR-Array analysis of CTEPH-ECs and Control-ECs. The fold regulation of the mRNA expression of CTEPH-ECs in comparison to Control-ECs from the angiogenesis PCR array (Qiagen, Human Angiogenesis [PAHS-024Z]). The genes that showed significantly increased or decreased expression (*p* < 0.05) are listed. The error bar represents the 95% confidence interval. Abbreviation: ANGPTL4; Angiopoietin-like 4, CTGF; Connective tissue growth factor, HGF; Hepatocyte growth factor, MMP14; Matrix metallopeptidase 14, SERPINE1; Serpin peptidase inhibitor 1 (plasminogen activator inhibitor-1), TYMP; Thymidine phosphorylase

The PCR array revealed that the RNA expression of HGF in CTEPH-ECs was higher than that in the control-ECs (Fold regulation 22.60, *p* = 0.016) (Fig. 3). Entire data of the PCR array is presented in Additional file 1. In response to this result, we also evaluated the mRNA expression of c-MET, RhoA, STAT3, ROCK1, ROCK2, COX-2 by qPCR analysis. CTEPH-ECs had slight increased expression of these molecules although there was no statistical difference (data not shown).

### Inhibition of HGF and Met *in vitro*

**Fig. 4:**
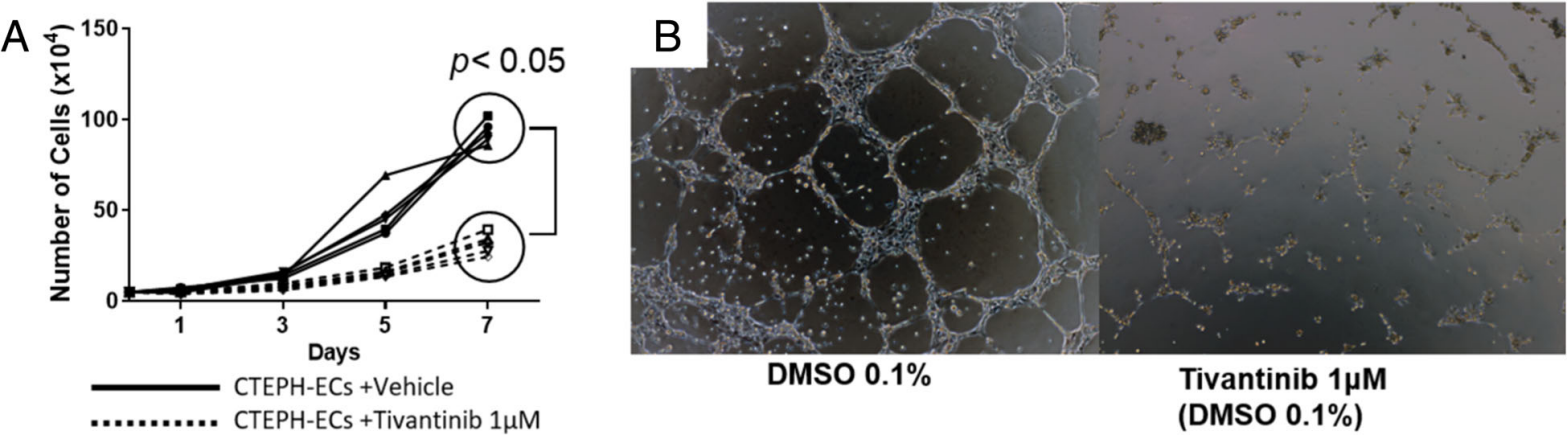
The assessment of the HGF/Met signal inhibition in CTEPH-ECs. **a** Cell culture in a 6-mm fibronectin-coated Petri dish, **b** Three dimensional culture. A selective HGF/Met inhibitor, tivantinib, suppressed cellular proliferation, and clearly inhibited tube formation

The addition of 1 μmol/L of tivantinib to the medium significantly suppressed the cellular proliferation (day 7, *p* = 0.0079) (Fig. 4a) and also appeared to suppress the tube formation of CTEPH-ECs (Fig. 4b).

### *In vivo* expression of HGF and Met

**Fig. 5:**
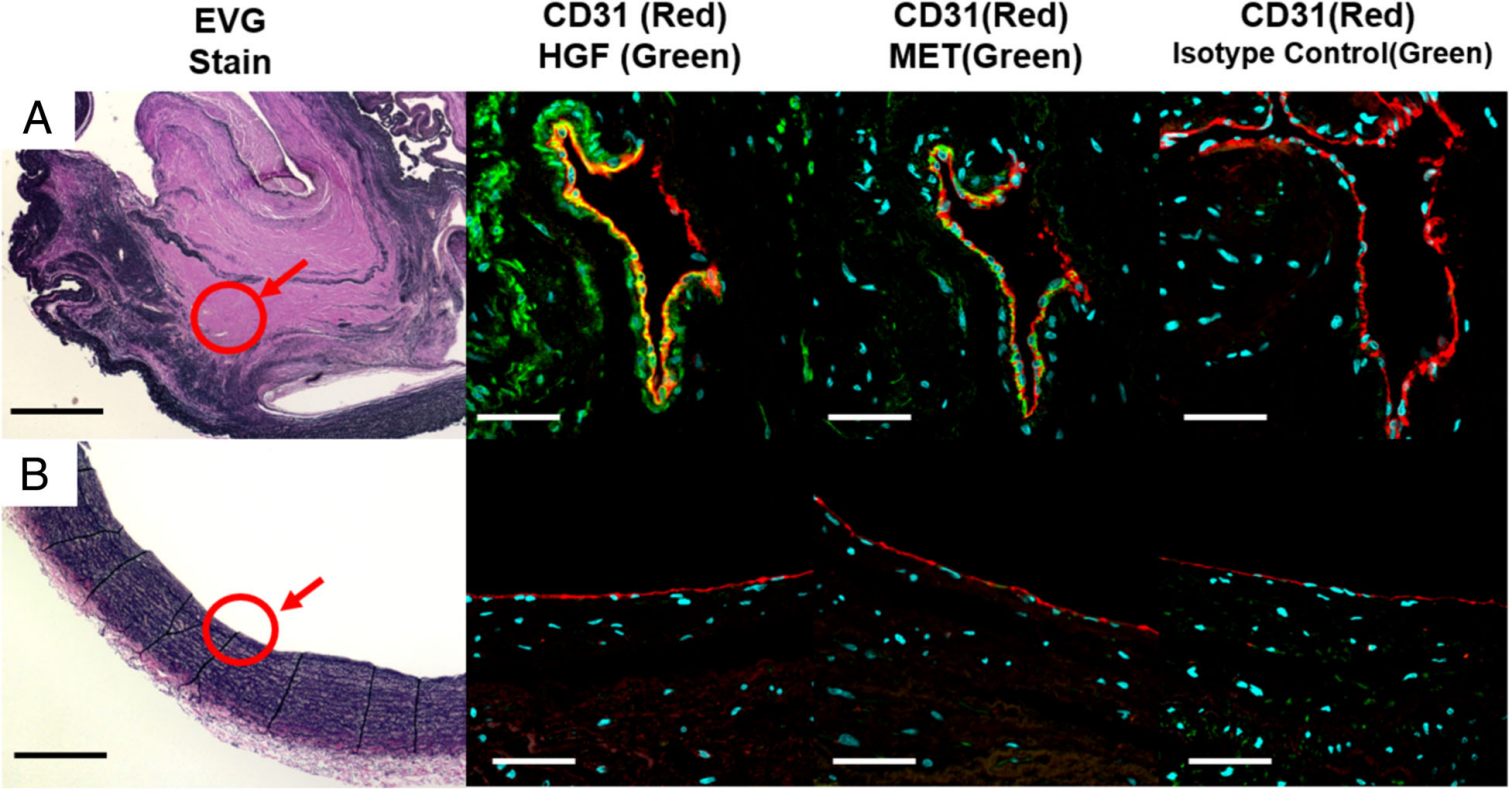
The immunohistochemical analysis of (**a**) thrombus vessels in pulmonary endarterectomy specimens and (**b**) control pulmonary arteries. The internal lumen of the thrombus vessels of the pulmonary endarterectomy specimens were positive for HGF and Met. Black Bar = 500 μm, White Bar = 50 μm

**Fig. 6:**
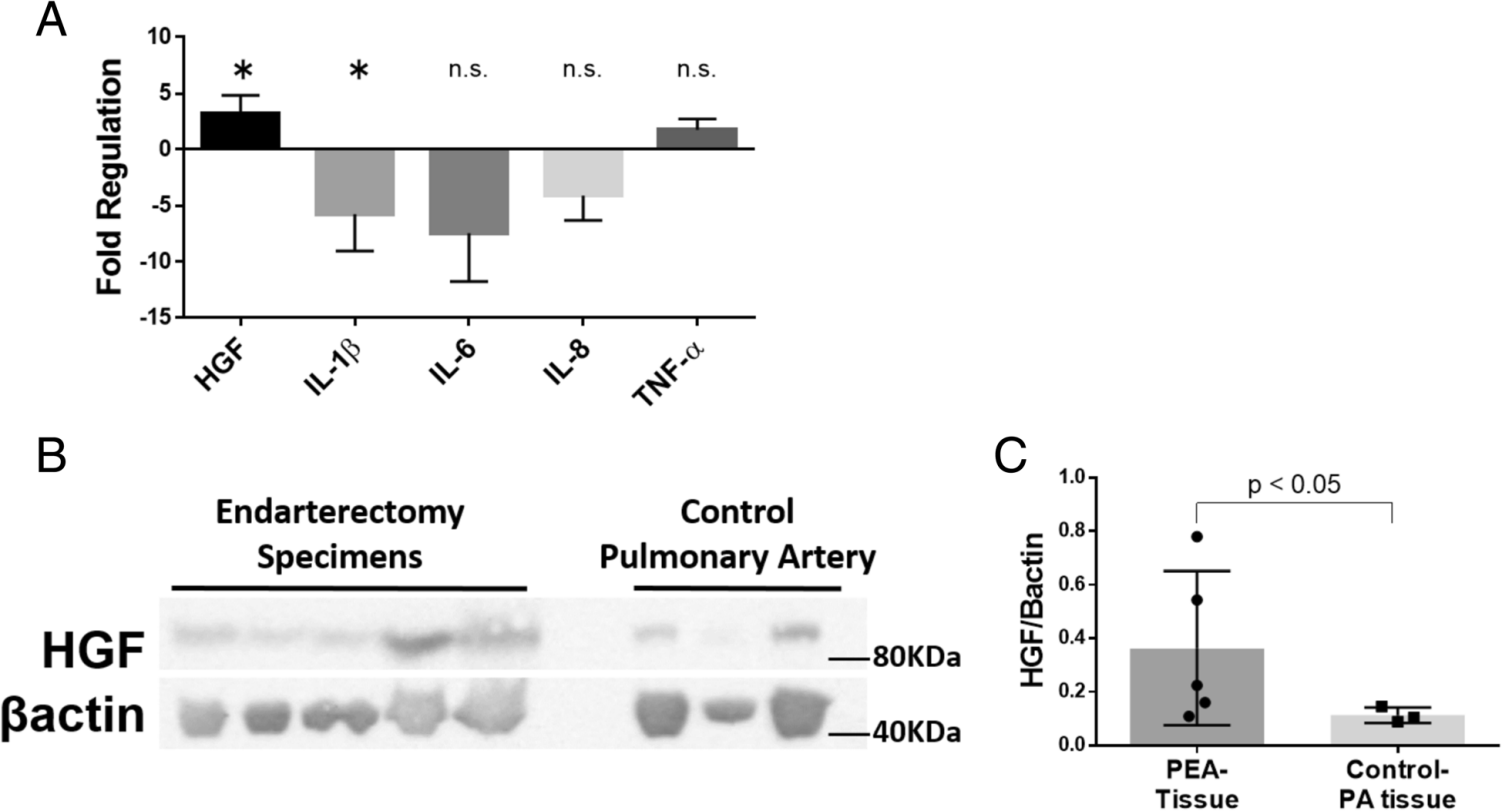
**a** PCR-Array analysis compairing the mRNA expression of HGF and inflammatory cytokines between pulmonary endarterectomy tissues and control pulmonary arteries. *: *p* < 0.05. The error bar represents the 95% confidence interval. Abbreviation: HGF; Hepatocyte Growth Factor, IL-1β; Interleukin-1β, IL-6; Interleukin-6, IL-8; Interleukin-8, TNF-α; Tumor Necrosis Factor-α. **b** The Western blotting of proteins extracted from pulmonary endarterectomy specimens and control pulmonary arteries. **c** The band signal strength of HGF is expressed as the ratio to beta-actin. The error bar represents the standard deviation. The expression of HGF protein from the pulmonary endarterectomy specimens was higher than that from the control pulmonary arteries

Immunohistochemical analysis of PEA specimens revealed that HGF and its receptor Met were expressed on the surface of thrombus vessels (Fig. 5a), while the internal lumens of the control pulmonary arteries were rarely positive for HGF and Met (Fig. 5b). The mRNA expression of HGF was higher in the PEA specimens, however, there were not significant mRNA upregulations of IL-1β, IL-6, TNF-αin PEA specimens (Fig. 6a). The HGF expression in extracted proteins from PEA specimens was also higher than that from the control pulmonary arteries (Fig. 6b, c).

### The serum HGF level

**Fig. 7:**
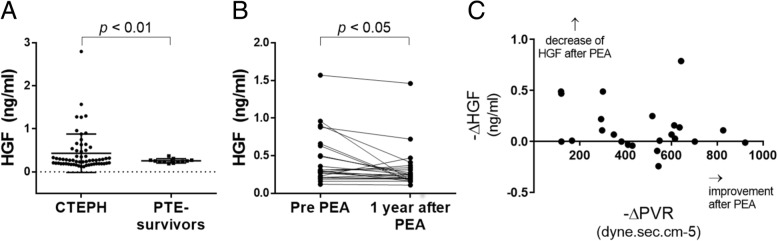
The serum levels of HGF in chronic thromboembolic pulmonary hypertension (CTEPH) patients (*n* = 61) and pulmonary thromboembolism (PTE) survivors without evidence of pulmonary hypertension (control group, *n* = 12). **a** The HGF serum level in the CTEPH group was higher than that in the control group (0.43 ± 0.25 ng/ml, 0.25 ± 0.04 ng/ml, respectively, *p* = 0.003). **b** HGF levels 1 year after PEA was also determined in those 23 patients. There was slight decrease of serum HGF level after PEA. **c** The difference between pre and post serum HGF level did not correlate with the improvement degree of PVR

Serum samples were collected from 61 additional CTEPH patients (include the patients who donated tissues) and 12 PTE survivors without evidence of pulmonary hypertension. The characteristics of these patients are listed in Table 1. The HGF serum level in the CTEPH group was higher than that in the control group (0.43 ± 0.44, vs 0.25 ± 0.04, *p* = 0.003) (Fig. 7a). In the CTEPH group, there was no correlation between the serum HGF level and the hemodynamic parameters (pulmonary artery pressure/resistance, the cardiac output, the oxygen level, and the plasma brain natriuretic peptide level) (data not shown). HGF levels 1 year after PEA was also determined in those 23 patients. There was slight decrease of serum HGF level (Pre PEA HGF; 0.44 ± 0.34 ng/ml, Post PEA HGF; 0.31 ± 0.28 ng/ml, *p* = 0.013) after PEA (Fig. 7b), while remarkable decrease of PVR (Pre PVR; 782.2 ± 254.0 dyne.sec.cm^− 5^, Post PVR; 317.4 ± 132.0 dyne.sec.cm^− 5^, *p* <  0.0001). The difference between pre and post serum HGF level did not correlate with the improvement degree of PVR. (Fig. 7c).

**Table 1:** Basic characteristics of patients whose blood sample were collected for the analysis of serum HGF in Fig. 7a

	CTEPH group	PTE survivors (Control group)	*p* value
Number of patients	61	12	
Age (y)	62.1 ± 10.6	71.0 ± 12.4	< 0.01
Sex (M:F)	14:47	0:12	n.s.
Systolic blood pressure (mmHg)	121.9 ± 21.5	135.4 ± 10.8	< 0.05
Diastolic blood pressure (mmHg)	73.9 ± 14.3	71.2 ± 10.0	n.s.
Total cholesterol (mg/dl)	192.6 ± 33.8	197.5 ± 24.9	n.s.
ALT (U/l)	25.9 ± 10.2	24.5 ± 6.1	n.s
AST (U/l)	20.5 ± 11.7	19.9 ± 6.93	n.s.
ɤ-GTP (U/l)	48.6 ± 49.9	39.5 ± 49.8	n.s.
C-Reactive Protein (mg/dl)	0.18 ± 0.33	0.11 ± 0.16	n.s.
BNP (pg/ml)	147.2 ± 264.5	40.1 ± 34.4	n.s.
Disease duration (month)^a^	32.1 ± 36.6	89.4 ± 42.6	*<* 0.01
Mean Pulmonary artery pressure (mmHg)	41.8 ± 10.3	–	–
Pulmonary artery resistance (dyne.sec.cm^− 5^)	661.6 ± 287.7	–	–
Cardiac Index (L/min/m^2^)	2.74 ± 0.68	–	–
NYHA(I:II:III:IV)	0:42:18:1	–	–

Data are presented as either mean ± SD or actual value

^a^Time from the onset of symptom to the blood sampling

Additionally, plasma levels of representative inflammatory and angiogenic molecules, which were associated with HGF (IL-1β, TNF-α, VEGF-A, AngiotensinII), were measured in 26 CTEPH patients and 12 PTE survivors from the above patients. However, there was no significant difference in plasma levels of these molecule between the two groups (Additional file 2).

## Discussion

**Fig. 8:**
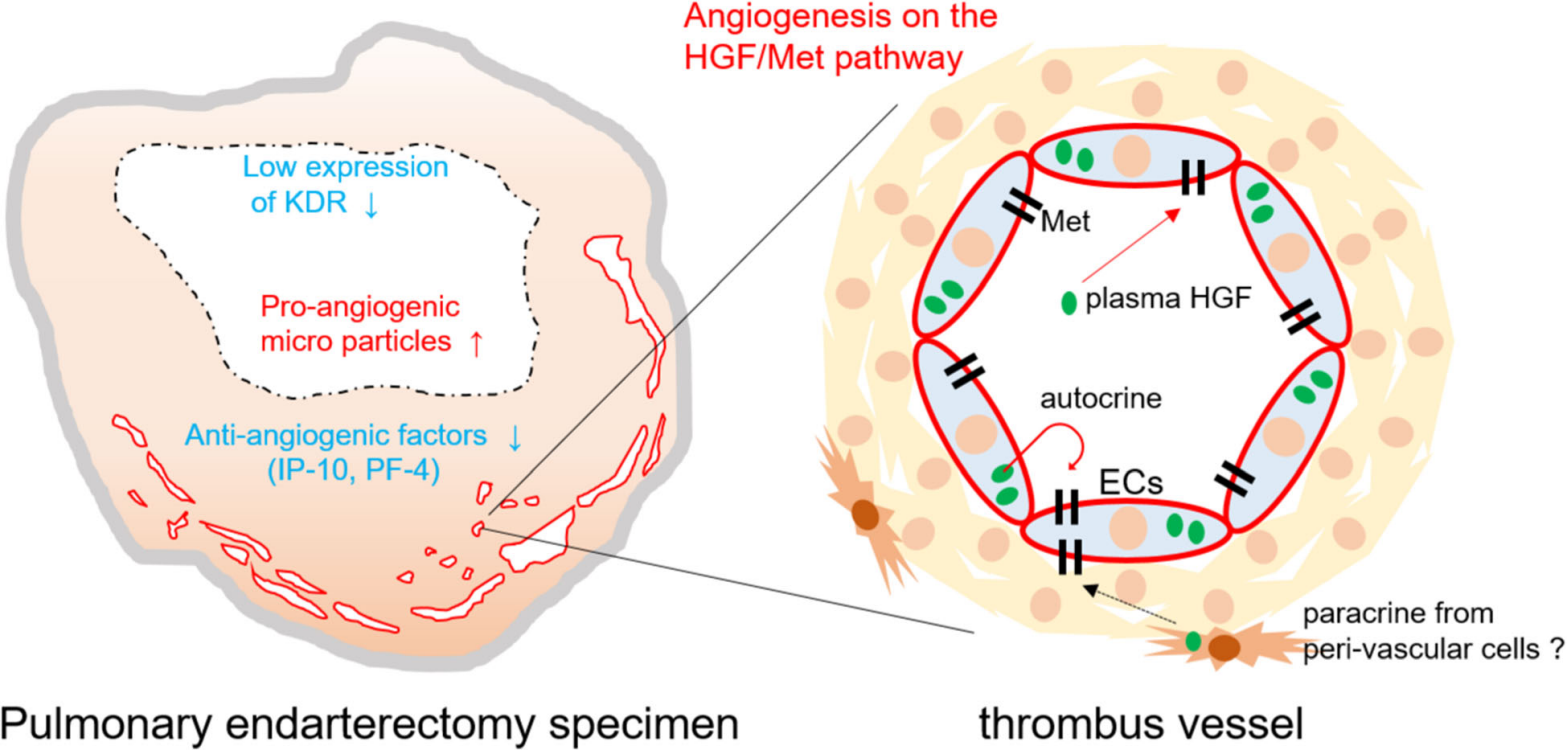
The hypothesized etiology of insufficient angiogenesis in pulmonary endarterectomy specimens. Insufficient angiogenesis in the organized thrombi might consist of complex conflicts between anti-angiogenic factors (decreased mRNA expression of angiogenetic factor like KDR, loscal anti-angiogenic cytokines) and pro-angiogenic factors (plasma microparticles). The finding of pro-angiogenic factors in the thrombus vessels in the present study (endothelial cells with high angiogenic potential) may represent the latter

Here we showed that CTEPH-ECs had a greater proliferative and angiogenic potential than control-ECs (Fig. 2a, c-d). We also showed that the expression levels of HGF were higher in CTEPH-ECs (Fig. 3), and that the inhibition of HGF action suppressed the proliferation and angiogenesis of CTEPH-EC (Fig. 4). Each cell line’s permeability was less in CTEPH-ECs than in Control-ECs, which also suggests the higher HGF activity [13] in CTEPH-ECs in vitro (Fig. 2c). In vivo experiments and immunohistochemical staining demonstrated the expression of HGF in the thrombus vessel lumens of PEA specimens (Fig. 5), while RT-PCR and western blotting demonstrated that the expression of HGF was higher in PEA specimens than in the control pulmonary arteries (Fig. 6). We also demonstrated the expression of Met (HGF receptor) on the surface of thrombus vessels. In the clinical setting, we showed that CTEPH patients had higher concentrations of serum HGF in comparison to PTE survivors without evidence of pulmonary hypertension (Fig. 7), although there were no correlation between serum HGF concentration and EC phenotype in vitro described in Figs. 2, 3. Our results showed that there were ECs with high angiogenic potential, which was driven at least in part by increased activity of the autocrine, paracrine and endocrine HGF-Met signaling pathway in the thrombus vessels of the PEA specimens (Fig. 8).

There are several basic reports about the impairment of angiogenesis in CTEPH patients. Alias et al. reported that vascular endothelial growth factor receptor-2 (KDR) had an important role in the resolution of venous thrombi, and that the mRNA expression of KDR in white thrombi was reduced in comparison to that in the non-thrombosed pulmonary arteries [4]. Zabini et al. reported that homogenized PEA specimen contained several cytokines that inhibited angiogenesis [14]. On the other hand, Belik et al. reported that the plasma microparticles of CTEPH patients facilitated angiogenesis in human pulmonary artery ECs [15]. These data suggested that deficient angiogenesis in organized thrombi might be associated with an imbalance between anti-angiogenic factors (the decreased mRNA expression of angiogenetic factor-like KDR and local anti-angiogenic cytokines) and pro-angiogenic factors (plasma microparticles). Our result that ECs in PEA specimen possessed high angiogenic potential may reflect the involvement of pro-angiogenic factors.

VEGF gene therapy has previously been reported to enhance venous thrombus recanalization and resolution [16, 17], and the endothelial cell-specific deletion of KDR abated the formation of vessel thrombosis and inhibited the resolution of thrombosis [4]. In the clinical setting, the findings of low levels of angiogenesis [3] and a small number of recanalized vessels [18] in PEA specimen predicts a poor prognosis in CTEPH patients. Complementing the above reports, the high angiogenic potential associated with HGF described here in the CTEPH-ECs may point towards a positive role in the resolution of thrombosis. In addition, numerous preclinical studies have recently examined the use of HGF as a pro-angiogenic and cardio-protective agent in the treatment of myocardial infarction, heart failure, and limb ischemia [7]. Together with our results, the targeted proper induction of HGF or HGF mimetics, which may lead to angiogenesis, may have a positive role in the treatment of obstructive and/or stenotic pulmonary vascular lesions in CTEPH patients.

Our pathobiological investigations also demonstrated that the internal surface of PEA specimen was rarely positive for CD31 (Additional file 3). This may suggest that most parts of the endothelial cell layers of the neointima were damaged before the PEA procedure. Furthermore, it suggests that the isolated CTEPH-ECs might have been derived from the microvascular ECs within the thrombus vessels, which may differ from the ECs on the internal surface of thrombus. If so, the pro-angiogenic expression of mRNA in white thrombi could depend on the number of thrombus vessels in the specimen, which was difficult to estimate without a pathological analysis of the same specimen. This hypothesis may be able to explain the difference between our results and the results of Alias et al. [4], which showed that the angiogenesis in white thrombi was deficient in comparison to the non-thrombosed pulmonary artery. Although, and this is a speculation, if the neointima was damaged prior to PEA, the ECs of the damaged neointima might have low angiogenic potential and might play some role in the organization of thrombosis.

The mechanism of HGF production in PEA tissue is undeterminded in our study. It is reported that HGF is secreted by stromal cells when they are stimulated by IL-1, IL-6, TNF-α [19], and that the levels of inflammatory cytokines (such as IL-6) are upregulated in the PEA tissues [20] and serum [21]. So we had assumed that the inflammatory cytokines in PEA specimens and the serum may stimulate ECs to secrete HGF. In this study, however, there is no increase of these inflammatory cytokines mRNA in the PEA specimens (Fig. 6a) and of these protein expression in serum (Additional file 2). Although it remains unknown whether the relationship between HGF protein and these inflammatory cytokines, increased expression of HGF mRNA and protein has been confirmed in patients with CTEPH. It is supposed that there might be other stimulators for HGF production instead of these cytokines.

Actually, it is not sure how an increased expression of serum HGF has a role in the development of organized thombus. However, it is reported that the serum HGF levels increase in patients with venous thrombosis [22] [23], pulmonary hypertension [24] as well as older age [25] and hypertension [26]. In this study, we showed that CTEPH patients had higher concentrations of serum HGF despite the younger age and lower systolic blood pressure in comparison to PTE survivors, while the decrease of HGF after successful PEA was slight in operated patients. It is currently unclear whether the increased serum level of HGF is a consequence of thrombosis itself or of other factors that are involved in the cardiovascular burden including pulmonary hypertension, but further investigation may provide us the unknown information on the etiology of PH.

The present study has several limitations. First, the number of CTEPH patients and control subjects was extremely small, and the differences between the CTEPH-ECs and the Control-ECs may not reflect the entire disease etiology. In addition, regarding histological analysis, we could not see the “whole thrombus”, but only the part of the removed specimens. There could be a heterogeneity in PEA specimens and this could have introduced a selection bias. Second, passaged cells in vitro assume a different phenotype compared with ECs in vivo. EGM medium which contains several growth factors (including VEGF and FGF) was used to isolate the ECs; this could have influenced the results of comprehensive PCR-array analysis of CTEPH-ECs in Fig. 3, especially about the molecules associated with VEGF and FGF. Third, there are likely complex interactions between ECs and other cells (fibroblast, smooth muscle cells, and others) in organized thrombi which are not occurring in EC monocultures. Such interactions need to be considered when interpreting our results. Forth, although it has been reported that there are stem-like cells in PEA specimens [27] and it has been speculated that those cells might affect angiogenesis of thrombus vessels; here we did not investigate stem-like cells. Despite those limitations, however, we believe that a detailed comparison between the CTEPH-ECs and the Control-ECs, which were isolated under the identical conditions, may be helpful for understanding the disease etiology and the development of novel targeted therapies for the disease.

## Conclusions

The results of our study demonstrate that there are ECs with pro-angiogenetic character and high expression of HGF in PEA specimens. It remains to be investigated how these results relate to chronic thromboembolus formation, however, further investigations focusing on HGF/ Met may provide novel diagnostic and therapeutic tools that may be used in the treatment of patients with CTEPH.

## Additional files

Additional file 1: The whole data of PCR-array analysis of CTEPH-ECs. The whole data of PCR-array analysis, which compaired the mRNA expression of CTEPH-ECs and Control-ECs (Fig. 3). (DOCX 21 kb)

Additional file 2: The serum levels of representative inflammatory and angiogenic cytokines in chronic thromboembolic pulmonary hypertension (CTEPH) patients (*n* = 26) and pulmonary thromboembolism (PTE) survivors without evidence of pulmonary hypertension (control group, *n* = 12). A) Interleukin-1β, B) Tumor Necrosis Factor-α, C) Vascular Endothelial Growth Factor-A, D)Angiotensin-2. There are no significant differences between two groups in these cytokine levels. (PDF 24 kb)

Additional file 3: CD31 staining of pulmonary endarterectomy (PEA) specimens. A~D): Immunohistochemical staining of CD31 in PEA specimens from different CTEPH patients. The thrombus vessels were clearly positive for CD31, while the internal surface of PEA specimens were rarely positive for CD31. Bar = 1 mm. (PDF 2298 kb)

## Abbreviations

CTEPH: Chronic thromboembolic pulmonary hypertension
HGF: Hepatocyte growth factor
Met: Mesenchymal-epithelial transition factor
