# Supplementary material for: Endothelial cells from pulmonary endarterectomy specimens possess a high angiogenic potential and express high levels of hepatocyte growth factor
Source: BMC Pulm Med. 2018 Dec 29;18:197. doi: 10.1186/s12890-018-0769-3 (PMC6310963; doi:10.1186/s12890-018-0769-3)
Supplement: Supplementary file 1 — The whole data of PCR-array analysis of CTEPH-ECs. The whole data of PCR-array analysis, which compaired the mRNA expression of CTEPH-ECs and Control-ECs (Fig. 3). (DOCX 21 kb) [file 12890_2018_769_MOESM1_ESM.docx]

**Additional file 1** The whole data of PCR-array analysis compairing the mRNA expression of CTEPH-ECs and Control-ECs

| **Symbol** | **gene name** | **Fold Regulation** | ***p-value*** |
| --- | --- | --- | --- |
| AKT1 | V-akt murine thymoma viral oncogene homolog 1 | -1.3533 | 0.233981 |
| ANG | Angiogenin, ribonuclease, RNase A family, 5 | 3.2316 | 0.207345 |
| ANGPT1 | Angiopoietin 1 | 2.6882 | 0.262957 |
| ANGPT2 | Angiopoietin 2 | 8.3246 | 0.173792 |
| ANGPTL4 | Angiopoietin-like 4 | -4.5116 | 0.000447 |
| ANPEP | Alanyl (membrane) aminopeptidase | -1.3657 | 0.575866 |
| ADGRB1 | Brain-specific angiogenesis inhibitor 1 | 6.2962 | 0.293352 |
| CCL11 | Chemokine (C-C motif) ligand 11 | 5.6064 | 0.307184 |
| CCL2 | Chemokine (C-C motif) ligand 2 | 1.74 | 0.360038 |
| CDH5 | Cadherin 5, type 2 (vascular endothelium) | -1.6555 | 0.419444 |
| COL18A1 | Collagen, type XVIII, alpha 1 | -2.5361 | 0.229039 |
| COL4A3 | Collagen, type IV, alpha 3 (Goodpasture antigen) | 5.03 | 0.295694 |
| CTGF | Connective tissue growth factor | 1.822 | 0.018045 |
| CXCL1 | Chemokine (C-X-C motif) ligand 1 (melanoma growth stimulating activity, alpha) | 1.0844 | 0.651107 |
| CXCL10 | Chemokine (C-X-C motif) ligand 10 | 6.1111 | 0.302629 |
| CXCL5 | Chemokine (C-X-C motif) ligand 5 | 5.5818 | 0.19888 |
| CXCL6 | Chemokine (C-X-C motif) ligand 6 (granulocyte chemotactic protein 2) | 1.3917 | 0.846767 |
| CXCL9 | Chemokine (C-X-C motif) ligand 9 | 8.1475 | 0.328294 |
| EDN1 | Endothelin 1 | 1.9768 | 0.13652 |
| EFNA1 | Ephrin-A1 | 1.1577 | 0.420893 |
| EFNB2 | Ephrin-B2 | 1.2194 | 0.483936 |
| EGF | Epidermal growth factor | 6.2782 | 0.276795 |
| ENG | Endoglin | 1.0805 | 0.639473 |
| EPHB4 | EPH receptor B4 | -1.5831 | 0.380761 |
| ERBB2 | V-erb-b2 erythroblastic leukemia viral oncogene homolog 2, neuro/glioblastoma derived oncogene homolog | -1.1408 | 0.90594 |
| F3 | Coagulation factor III (thromboplastin, tissue factor) | 5.173 | 0.328863 |
| FGF1 | Fibroblast growth factor 1 (acidic) | 1.5825 | 0.584751 |
| FGF2 | Fibroblast growth factor 2 (basic) | 1.4402 | 0.459847 |
| FGFR3 | Fibroblast growth factor receptor 3 | -1.054 | 0.58838 |
| FIGF | C-fos induced growth factor (vascular endothelial growth factor D) | 5.001 | 0.112737 |
| FLT1 | Fms-related tyrosine kinase 1 (vascular endothelial growth factor/vascular permeability factor receptor) | 1.6227 | 0.218226 |
| FN1 | Fibronectin 1 | -1.0549 | 0.716175 |
| HGF | Hepatocyte growth factor (hepapoietin A; scatter factor) | 22.6016 | 0.016096 |
| HIF1A | Hypoxia inducible factor 1, alpha subunit (basic helix-loop-helix transcription factor) | 3.0429 | 0.098405 |
| HPSE | Heparanase | 3.3679 | 0.150165 |
| ID1 | Inhibitor of DNA binding 1, dominant negative helix-loop-helix protein | 1.3619 | 0.45578 |
| IFNA1 | Interferon, alpha 1 | 1.3371 | 0.359181 |
| IFNG | Interferon, gamma | 4.7731 | 0.298019 |
| IGF1 | Insulin-like growth factor 1 (somatomedin C) | 1.6475 | 0.299452 |
| IL1B | Interleukin 1, beta | 5.7196 | 0.306791 |
| IL6 | Interleukin 6 (interferon, beta 2) | 1.6113 | 0.214814 |
| CXCL8 | Interleukin 8 | 1.1901 | 0.429518 |
| ITGAV | Integrin, alpha V (vitronectin receptor, alpha polypeptide, antigen CD51) | 1.35 | 0.379507 |
| ITGB3 | Integrin, beta 3 (platelet glycoprotein IIIa, antigen CD61) | 1.4342 | 0.305337 |
| JAG1 | Jagged 1 | -1.0121 | 0.798983 |
| KDR | Kinase insert domain receptor (a type III receptor tyrosine kinase) | 1.2946 | 0.360891 |
| LECT1 | Leukocyte cell derived chemotaxin 1 | 5.0998 | 0.31596 |
| LEP | Leptin | 3.678 | 0.317638 |
| MDK | Midkine (neurite growth-promoting factor 2) | 3.7184 | 0.187203 |
| MMP14 | Matrix metallopeptidase 14 (membrane-inserted) | -2.9077 | 0.037012 |
| MMP2 | Matrix metallopeptidase 2 (gelatinase A, 72kDa gelatinase, 72kDa type IV collagenase) | -1.0898 | 0.862163 |
| MMP9 | Matrix metallopeptidase 9 (gelatinase B, 92kDa gelatinase, 92kDa type IV collagenase) | 4.9281 | 0.289217 |
| NOS3 | Nitric oxide synthase 3 (endothelial cell) | -1.4512 | 0.473312 |
| NOTCH4 | Notch 4 | 1.5444 | 0.310281 |
| NRP1 | Neuropilin 1 | 1.5764 | 0.301553 |
| NRP2 | Neuropilin 2 | -1.5773 | 0.508504 |
| PDGFA | Platelet-derived growth factor alpha polypeptide | 1.0292 | 0.967576 |
| PECAM1 | Platelet/endothelial cell adhesion molecule | 2.2734 | 0.241548 |
| PF4 | Platelet factor 4 | 12.67 | 0.391641 |
| PGF | Placental growth factor | -1.3483 | 0.183653 |
| PLAU | Plasminogen activator, urokinase | -3.0497 | 0.223277 |
| PLG | Plasminogen | 6.661 | 0.299362 |
| PROK2 | Prokineticin 2 | 5.2453 | 0.302496 |
| PTGS1 | Prostaglandin-endoperoxide synthase 1 (prostaglandin G/H synthase and cyclooxygenase) | 2.5168 | 0.406905 |
| S1PR1 | Sphingosine-1-phosphate receptor 1 | 2.0504 | 0.216114 |
| SERPINE1 | Serpin peptidase inhibitor, clade E (nexin, plasminogen activator inhibitor type 1), member 1 | -1.354 | 0.001891 |
| SERPINF1 | Serpin peptidase inhibitor, clade F (alpha-2 antiplasmin, pigment epithelium derived factor), member 1 | -1.2364 | 0.91295 |
| SPHK1 | Sphingosine kinase 1 | -2.5484 | 0.117075 |
| TEK | TEK tyrosine kinase, endothelial | 1.3757 | 0.409953 |
| TGFA | Transforming growth factor, alpha | 1.2707 | 0.813092 |
| TGFB1 | Transforming growth factor, beta 1 | 1.1345 | 0.519314 |
| TGFB2 | Transforming growth factor, beta 2 | 1.2347 | 0.49163 |
| TGFBR1 | Transforming growth factor, beta receptor 1 | 1.3496 | 0.499164 |
| THBS1 | Thrombospondin 1 | 1.0196 | 0.810766 |
| THBS2 | Thrombospondin 2 | 45.2902 | 0.272211 |
| TIE1 | Tyrosine kinase with immunoglobulin-like and EGF-like domains 1 | -1.482 | 0.804098 |
| TIMP1 | TIMP metallopeptidase inhibitor 1 | 3.4612 | 0.227445 |
| TIMP2 | TIMP metallopeptidase inhibitor 2 | 1.8152 | 0.281538 |
| TIMP3 | TIMP metallopeptidase inhibitor 3 | 2.3104 | 0.743431 |
| TNF | Tumor necrosis factor | -1.3888 | 0.814023 |
| TYMP | Thymidine phosphorylase | -6.6577 | 0.016648 |
| VEGFA | Vascular endothelial growth factor A | 1.9814 | 0.264379 |
| VEGFB | Vascular endothelial growth factor B | 2.673 | 0.139754 |
| VEGFC | Vascular endothelial growth factor C | 3.0481 | 0.246727 |

The whole data of PCR-array analysis, which compaired the mRNA expression of CTEPH-ECs and Control-ECs (figure 3).
