# Supplementary figures and images for: Endothelial cells from pulmonary endarterectomy specimens possess a high angiogenic potential and express high levels of hepatocyte growth factor
Source: BMC Pulm Med. 2018 Dec 29;18:197. doi: 10.1186/s12890-018-0769-3 (PMC6310963; doi:10.1186/s12890-018-0769-3)

A)

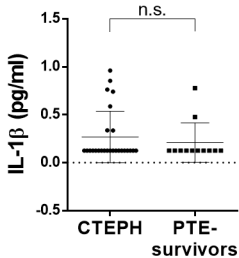

B)

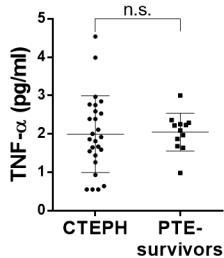

C)

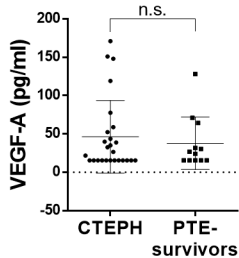

D)

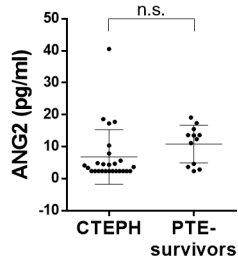

Supplement: Supplementary file 2 — The serum levels of representative inflammatory and angiogenic cytokines in chronic thromboembolic pulmonary hypertension (CTEPH) patients (n = 26) and pulmonary thromboembolism (PTE) survivors without evidence of pulmonary hypertension (control group, n = 12). A) Interleukin-1β, B) Tumor Necrosis Factor-α, C) Vascular Endothelial Growth Factor-A, D)Angiotensin-2. There are no significant differences between two groups in these cytokine levels. (PDF 24 kb) [file 12890_2018_769_MOESM2_ESM.pdf]

**A)**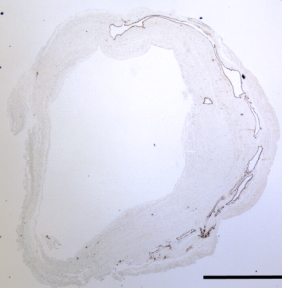**B)**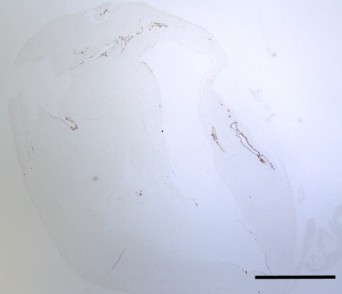**C)**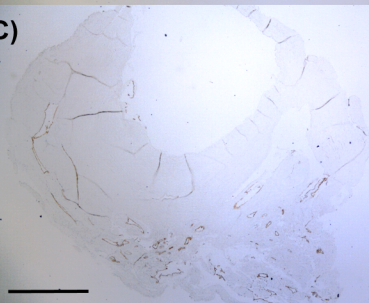**D)**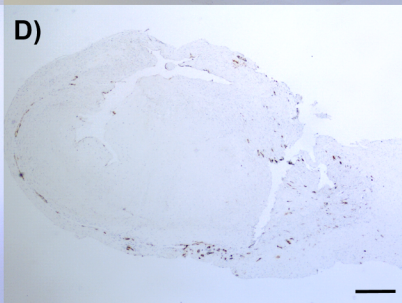

Supplement: Supplementary file 3 — CD31 staining of pulmonary endarterectomy (PEA) specimens. A~D): Immunohistochemical staining of CD31 in PEA specimens from different CTEPH patients. The thrombus vessels were clearly positive for CD31, while the internal surface of PEA specimens were rarely positive for CD31. Bar = 1 mm. (PDF 2298 kb) [file 12890_2018_769_MOESM3_ESM.pdf]
